# Impact of Automated Insulin Delivery Systems in Children and Adolescents with Type 1 Diabetes Previously Treated with Multiple Daily Injections: A Single-Center Real-World Study

**DOI:** 10.3390/medicina61091602

**Published:** 2025-09-05

**Authors:** Bruno Bombaci, Marco Calderone, Alessandra Di Pisa, Mariarosaria La Rocca, Arianna Torre, Fortunato Lombardo, Giuseppina Salzano, Stefano Passanisi

**Affiliations:** Department of Human Pathology and Developmental Age “G. Barresi”, University of Messina, 98122 Messina, Italy; marco.calderone@studenti.unime.it (M.C.); alessandra.dipisa@studenti.unime.it (A.D.P.); mariarosaria.larocca@studenti.unime.it (M.L.R.); trrrnn95p56f158l@studenti.unime.it (A.T.); fortunato.lombardo@unime.it (F.L.); gsalzano@unime.it (G.S.); stepassanisi@unime.it (S.P.)

**Keywords:** advanced hybrid closed loop, BMI, time in range, time in tight range, total daily dose

## Abstract

*Background and Objectives*: Automated insulin delivery (AID) systems represent a major advancement in type 1 diabetes (T1D) management, particularly in pediatric populations. However, real-world evidence comparing their effectiveness to conventional multiple daily injection (MDI) therapy in youth remains limited. This study aimed to evaluate the impact of transitioning from MDI therapy to AID systems on glycemic control in children and adolescents with T1D, and to explore potential differences based on baseline HbA1c levels and device type. *Materials and Methods*: In this single-center, retrospective observational study, 76 children and adolescents with T1D were evaluated before and after switching from MDI to either the Medtronic MiniMed™ 780G or Tandem t:slim X2™ Control-IQ system. Glycemic control was assessed using continuous glucose monitoring (CGM)-derived metrics at three time points: the last 15 days of MDI therapy (T0), 15 days after (T1), and 6 months after (T2) AID initiation. Statistical comparisons were conducted across time points and between subgroups stratified by baseline HbA1c and AID system. *Results*: Significant improvements in glycemic control were observed as early as 15 days after AID initiation, with sustained benefits at 6 months. Time in range (TIR) increased from 62.0% at baseline to 76.7% at 15 days and 75.8% at 6 months, and time in tight range (TITR) from 39.8% to 53.9% at T1 and 52.1% at T2 (both *p* < 0.001). Improvements were more pronounced in participants with higher baseline HbA1c (+16.9% for TITR and +22.3% for TIR). No significant differences in glycemic outcomes were observed between device groups, although algorithm-driven differences in insulin delivery patterns were noted. Total daily insulin dose and BMI increased significantly over time (*p* < 0.001 and *p* = 0.008, respectively). *Conclusions*: AID therapy leads to rapid and sustained improvements in glycemic control among youth with T1D, particularly in those with suboptimal baseline control. These benefits highlight the clinical value of AID systems, while also emphasizing the need for monitoring potential metabolic impacts.

## 1. Introduction

In recent years, technological advancements have significantly transformed the management of type 1 diabetes (T1D), especially in the pediatric and adolescent population. Automated insulin delivery (AID) systems have become a pivotal element in the standard of care, offering an integrated and adaptive method of insulin administration. These systems facilitate more consistent achievement of personalized glycemic targets, thereby reducing the daily management burden on families and caregivers and improving quality of life for children with T1D [1].

The introduction of hybrid closed loop (HCL) systems represents a crucial step toward the development of an artificial pancreas, a device designed to simulate the physiological functions of the endocrine pancreas. These systems integrate continuous glucose monitoring (CGM) with an insulin pump through an algorithm that automatically adjusts the insulin delivery based on glucose level variation. A notable feature of this system is its dynamic insulin sensitivity adjustment along with Predictive Low-Glucose Suspend (PLGS) functionality, enabling it to suspend basal insulin delivery during both hypoglycemia and when a rapid reduction in glycemic levels is signaled by the glucose sensor [2].

Introduced in 2017, the Medtronic MiniMed™ 670G (Medtronic, Northridge, CA, USA) was the first commercially available HCL system, approved for individuals aged over 7 years with T1D. This technological evolution has culminated in the next-generation Medtronic MiniMed 780G™ system (Medtronic, Northridge, CA, USA), which addresses several limitations of its predecessor. This system employs a proportional–integral–derivative (PID) algorithm in combination with fuzzy logic to optimize insulin delivery. This algorithm provides automated basal insulin adjustments at 5 min intervals, continuously adjusting insulin delivery based on real-time CGM (rtCGM), and automatic correction bolus delivery in cases of incipient hyperglycemia. The system also allows users to select personalized glycemic targets (100, 110, or 120 mg/dL), enhancing flexibility and control [3,4].

The Tandem t:slim X2™ insulin pump, integrated with Control-IQ technology (Tandem Diabetes Care, San Diego, CA, USA) and released in early 2020, is another AID system widely used by children and adolescents with T1D. This technology can be prescribed to individuals aged six years or older and employs a predictive algorithm that adjusts basal insulin delivery every 5 min according to glucose predictions 30 min ahead, with the goal of maintaining glucose levels within the treatment range of 112.5–160 mg/dL. When glucose levels are expected to exceed 180 mg/dL, the system delivers automatic correction boluses, while suspending insulin delivery when hypoglycemia (<70 mg/dL) is predicted. The algorithm adopts model predictive control (MPC) strategies to dynamically optimize insulin dosing. Additionally, the system also includes specialized modes such as “Sleep” and “Exercise” to tailor insulin delivery to different physiological states [5,6].

Several other systems using HCL technology are currently available in the global marketplace. These include sensor-augmented pumps such as the Omnipod5 automated mode (HypoProtect™) from Insulet and the Medtrum Nano, known as the TouchCare^®^ Nano Insulin Patch Pump, as well as other AID systems like the CamAPS FX DanaRS or YpsoPump (1). Additionally, over the past decade, a distinct branch of AID research has led to the emergence of the so-called Do-it-Yourself (DIY) insulin delivery systems [7].

Despite the increasing use of AID systems, real-world data on children and adolescents transitioning directly from multiple daily injection (MDI) therapy remain limited. Most available studies have either evaluated AID systems in already pump-treated patients or reported outcomes without distinguishing by prior treatment modality. Therefore, the impact of switching from conventional MDI to advanced AID systems in pediatric populations is not fully understood. Generating evidence in this field is clinically relevant, as a large proportion of children still initiate insulin therapy with MDI and may later be candidates for AID systems. Among the commercially available devices, the Medtronic MiniMed™ 780G (MM780G) and the Tandem t:slim X2™ with Control-IQ (CIQ) are the most widely used in the pediatric population. Both have demonstrated safety and efficacy in clinical trials, yet few studies have compared their performance in routine practice, particularly in youth transitioning from MDI. Our study aimed to address this gap by evaluating the real-world effectiveness of transitioning from MDI to AID therapy.

## 2. Materials and Methods

In this single-center, retrospective, observational study, we enrolled children and adolescents with T1D using the following AID systems: MM780G or CIQ. The study adhered to the principles outlined in the Declaration of Helsinki and received approval on 4 May 2023 by the local Ethics Committee (no. 39–23). Written informed consent was obtained from the patient(s) or their legal caregivers prior to the study procedures. Participants were recruited based on the following inclusion criteria: diagnosis of T1D according to the latest ISPAD guidelines [8], age ≤ 18 years, therapy with an AID system for at least 6 months at the time of recruitment, duration of CGM activity during MDI therapy > 70%, transition from MDI therapy to AID therapy, and informed parental consent to access cloud-based continuous glucose monitoring data. Exclusion criteria included skin issues preventing the use of the glucose sensor, pharmacological therapies or clinical conditions known to cause alterations in glucose levels, insulin treatment different from MDIs (i.e., first-generation AID systems, such as the MiniMed 670G™, other AID systems, or other insulin pumps) and glucose monitoring different from CGM during MDI therapy. The following clinical and demographic data were collected: age, biological sex, onset date, duration of disease at the start of AID therapy, anthropometric parameters, type of AID system, daily insulin requirement and its distribution between pre-prandial and basal insulin dose, mean HbA1c related to the last 6 months of MDI therapy and the first 6 months following the initiation of AID therapy.

Two-week-period CGM data were gathered using specific web-cloud platforms (CareLink^®^ Professional software, Glooko^®^Web, Dexcom^®^ Clarity and Freestyle^®^ Libreview) at specific time points: the last 15 days of MDI therapy (T0), 15 days after starting AID therapy (T1), and 6 months after starting AID therapy (T2). For each time point, the following CGM metrics were collected, if available: time in range (TIR, glucose levels between 70 and 180 mg/dL), time in tight range (TITR, glucose levels between 70 and 140 mg/dL), time below range (TBR) divided into TBR1 (TBR Level 1, Low: glucose levels between 54 and 69 mg/dL) and TBR2 (TBR Level 2, Very Low: glucose levels below 54 mg/dL), time above range (TAR) divided into TAR1 (TAR Level 1, High: glucose levels between 181 and 250 mg/dL) and TAR2 (TAR Level 2, Very High: Glucose levels above 250 mg/dL), Glycemia Risk Index (GRI), mean sensor glucose and standard deviation (SD), coefficient of variation (CV), and glucose management indicator (GMI). GRI was calculated according to the following formula: (3.0 × TBR2) + (2.4 × TBR1) + (1.6 × TAR2) + (0.8 × TAR1) [9]. GMIs were automatically derived from the respective CGM software platforms (https://carelink.medtronic.eu/login; https://eu.my.glooko.com/users/sign_in; https://clarity.dexcom.eu/; https://www.libreview.com/, accessed on 29 August 2025). While raw data harmonization was not possible, we ensured that all metrics were calculated according to standardized international consensus definitions, regardless of cloud-based platforms.

### Statistical Analysis

Numerical variables are expressed as mean ± standard deviation (SD). Categorical data are presented as counts and corresponding percentages. Descriptive statistics were computed for each time point of observation. Given that most continuous variables showed a normal distribution, verified through the Kolmogorov–Smirnov test, a parametric approach was employed. Changes in continuous variables over time (T0, T1, and T2) were analyzed using repeated-measures analysis of variance (ANOVA). For pairwise comparisons, the paired *t-*test was utilized, with Bonferroni correction applied to adjust the alpha level (α) in light of the three possible comparisons. The chi-squared (χ^2^) test was used to compare categorical variables across time points. TIR and TITR changes across the study period in different subgroups according to baseline HbA1c levels were assessed by using the paired *t-*test. Comparisons between subgroups based on device brand were conducted using unpaired *t-*tests for continuous variables and χ^2^ or Fisher’s exact tests, as appropriate, for categorical variables. Statistical analyses were carried out using SPSS version 22.0 (IBM Corp., Armonk, NY, USA) for Windows. A *p*-value < 0.05 was considered statistically significant.

## 3. Results

Our study population included 76 children and adolescents with T1D, with a predominance of males (64.5%). The mean age was 11.8 ± 3.9 years, and the mean duration of diabetes was 3.7 ± 3.2 years. Among the participants, 32 (42.2%) started AID therapy with the MM780G system, while 44 (57.8%) used the CIQ system. All participants who initiated the CIQ system were previously using a Dexcom^®^ sensor (Dexcom, Inc., San Diego, CA, USA), while those who transitioned to the MM780G system were monitoring glucose with a Freestyle^®^ sensor (Abbott Laboratories, Chicago, IL, USA). The mean HbA1c during the six months preceding AID initiation was 6.8%, ranging from 5.4% to 8.6%.

During the final two weeks of MDI therapy before AID initiation, only 30% of participants achieved TIR > 70% and 25% achieved TITR > 50%. The mean TIR was 62.0 ± 15.3%, TITR was 39.8 ± 13%, TAR was 34.8 ± 15.8% and TBR was 3.0 ± 3.6%. GRI was 43.0 ± 21.6, GMI was 7.2 ± 0.8%, and mean glucose levels were 163.4 ± 26.2 mg/dL. The mean baseline HbA1c was 6.8 ± 0.7%.

### 3.1. Analysis of Glucose Outcomes and Insulin Data Across the Study

During the first 15 days of AID therapy in automatic mode, there was a significant improvement in glycemic control, reflected by an increase in TIR (76.7 ± 8.4%; *p* < 0.001) and TITR (53.9 ± 10.4%; *p* < 0.001), along with a reduction in TAR (20.3 ± 8.5%; *p* < 0.001), TAR1 (16.4 ± 6.7%; *p* < 0.001), TAR2 (3.9 ± 3.5% *p* < 0.001), mean glucose (141.3 ± 11.9 mg/dL; *p* < 0.001) and CV (35.1% ± 6.1%; *p* = 0.005). GRI decreased to 24.0 ± 8.0 (*p* < 0.001), and GMI was 6.7 ± 0.3%.

**Figure 1:**
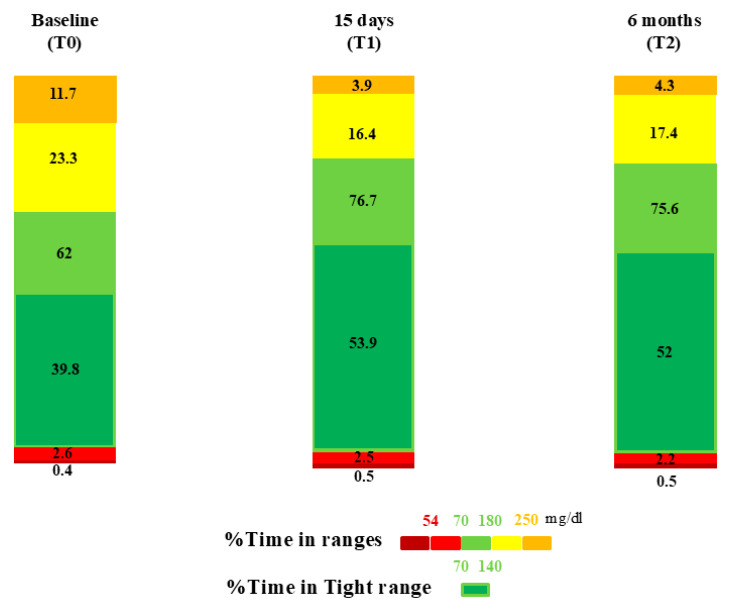
Comparison of CGM-derived metrics across the study period.

As shown in Table 1, all CGM metrics improved significantly at T2 compared to baseline. At six months, TIR was 75.8 ± 9.0%, TAR was 21.6 ± 9.6% (TAR1 17.4 ± 7.5%, TAR2 4.3 ± 3.9%) and TBR was 2.6 ± 2.5% (TBR1 2.2 ± 1.9, TBR2 0.5 ± 0.8%) (Figure 1). GMI was 6.8 ± 0.4%, mean glucose was 144.6 ± 14.8 mg/dL, and CV was 34.2 ± 6.1%. TITR reached 52.1 ± 10.5% and GRI was 27.4 ± 10.3. AID systems were used in automatic mode for 95% ± 6.2% of the time.

**Figure 2:**
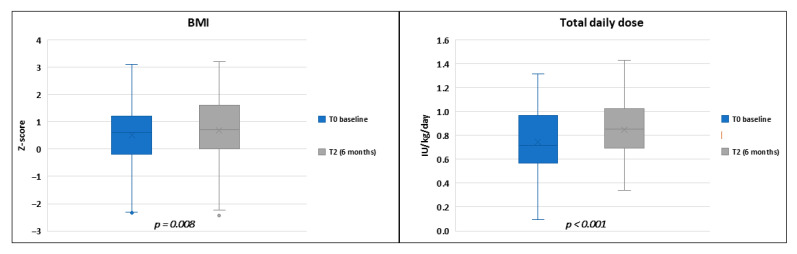
Box plots illustrating the differences in body mass index (BMI) and total daily dose of insulin between the initiation of automated insulin delivery therapy and the end of the follow-up study.

Total daily insulin dose increased significantly across the study period (0.75 ± 0.29 IU/kg at T0 vs. 0.85 ± 0.23 IU/kg at T2; *p* < 0.001). Similarly, BMI z-score also increased from 0.5 ± 1.2 SD at the baseline to 0.7 ± 1.2 SD at six months of follow-up (*p* = 0.008) (Figure 2).

### 3.2. Comparison of Clinical Data and Glucose Metrics Between Different Subgroups

**Figure 3:**
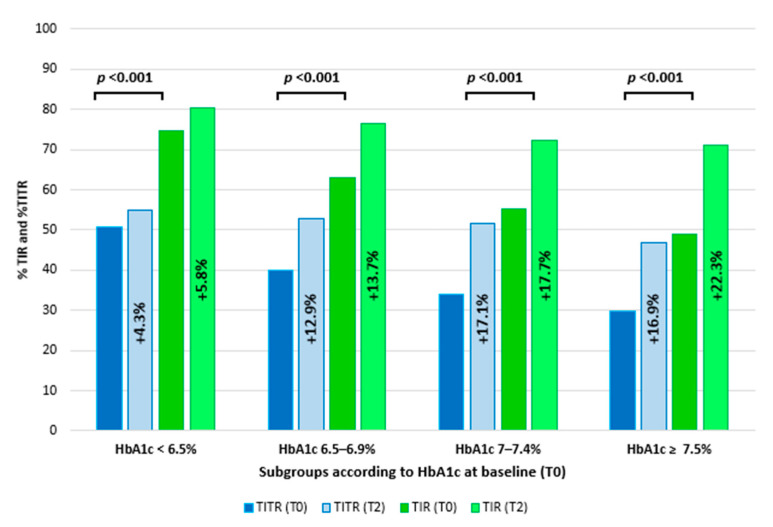
Graphical representation of time in range (TIR) and time in tight range (TITR) variations from T0 to T2 in different subgroups based on the baseline HbA1c levels. Both TIR and TITR significantly improved in all subgroups.

Glycemic improvement was analyzed across four subgroups based on baseline HbA1c levels: <6.5%, 6.5–6.9%, 7.0–7.4%, and ≥7.5%. As shown in Figure 3, TIR and TITR significantly increased from T0 to T2 in all subgroups (*p* < 0.001), with the greatest improvements observed among participants with higher baseline HbA1c. In particular, among participants with baseline HbA1c < 6.5%, TIR increased by 5.8% and TITR by 4.3%. In the subgroup with baseline HbA1c 6.5–6.9%, TIR improved from 62.9% to 76.6% and TITR from 40.0% to 52.9%. Participants with baseline HbA1c 7.0–7.5% showed increases of 17.1% in TIR and 17.7% in TITR. Finally, in those with baseline HbA1c > 7.5%, TIR rose from 48.9% to 71.2%, while TITR increased from 29.8% to 46.7%.

When stratified by device brand, no significant differences were observed in demographic and clinical characteristics (biological sex, age, disease duration) or anthropometric measures, including BMI z-score, at baseline.

Analysis of AGP-derived metrics revealed significant lower TBR (*p* = 0.015), TBR1 (*p* = 0.034), TBR2 (*p* = 0.031), and CV (*p* = 0.035) among MM780G users at T1. At six months, only TBR2 and CV remained significantly lower (*p* = 0.021 and *p* = 0.016, respectively), accompanied by a reduction in TAR2 (*p* = 0.019) in the MM780G group. No significant differences were observed in other key CGM metrics, including TIR, TITR, mean glucose, GRI, TAR and TAR1. Participants using the CIQ system had a lower percentage of automatic correction boluses and a higher percentage of basal insulin delivery (*p* < 0.001) (Table 2).

## 4. Discussion

In this real-world observational study, the transition from conventional MDI therapy to AID systems led to a significant improvement in glycemic control among children and adolescents with T1D. Specifically, we observed a higher percentage of time spent within both standard (70–180 mg/dL) and tighter (70–140 mg/dL) glucose target ranges, alongside a marked reduction in hyperglycemia. Notably, improvements in glycemic metrics, including reductions in both hypo- and hyperglycemic excursions, were evident as early as 15 days after AID initiation and remained stable over the six-month follow-up.

The superiority of AID systems over other therapeutic approaches has been well-documented in randomized controlled trials and observational studies [10,11,12]. These benefits related to their use include both clinical aspects, allowing the achievement of more stringent glycemic targets, and psychological aspects, leading to a lower perceived impact of diabetes on daily life for youth and their caregivers [13,14,15,16].

Our findings are consistent with previous pediatric studies evaluating the MM780 and CIQ systems separately. Specifically, sustained improvements in glycemic outcomes with the MM780G have been reported over follow-up periods of up to three years [17,18,19]. Likewise, large cohort studies support the safety and effectiveness of the CIQ system, showing significant reductions in severe hypoglycemia and diabetic ketoacidosis [6,20].

Few studies have directly compared these AID systems in pediatric populations [21,22]. While some authors have suggested better glycemic outcomes with the MM780G [23,24,25], our data did not reveal significant differences between the two devices in achieving key CGM-derived metrics (i.e., TIR, TITR, TAR, TBR or average glucose levels), which fell within the targets recommended by international guidelines [26,27]. However, we observed notable algorithm-driven differences: the CIQ group showed a higher proportion of basal insulin delivery, whereas the MM780G group had more automatic corrective boluses. These differences reflect their underlying algorithms. The MM780G uses a PID model capable of multiple boluses per hour, while the CIQ employs a MPC algorithm that prioritizes basal adjustments and limits to one automatic correction bolus per hour [28,29].

Notably, the greatest improvements in TIR and TITR at six months were detected in individuals who had suboptimal glycemic control and higher baseline HbA1c levels. This pattern, consistent with prior research, supports the theory that youth with high-risk or poorly controlled T1D may obtain the greatest benefit from AID therapy [30,31]. Specifically, Castorani et al. reported a 40% increase in mean TIR within two weeks of AID initiation in previously non-adherent adolescents [32]. Similarly, Boucsein et al. observed a mean HbA1c reduction of nearly 3% over three months in youth with suboptimal control and poor treatment [33].

Beyond glycemic outcomes, psychosocial and behavioral aspects represent a key dimension of AID therapy in pediatric populations. Although not directly assessed in our study, several recent investigations have reported improvements in treatment satisfaction, diabetes-related quality of life, and reductions in caregiver burden among youth using AID systems. Moreover, adherence to device use appears to be high in real-world pediatric cohorts, reflecting both the usability of these systems and the support provided by multidisciplinary diabetes teams.

We also observed a significant increase in total daily insulin dose and BMI over the study period. The metabolic impact of AID systems in youth with T1D remains debated. While some studies have reported a significant BMI increase following AID use [34], a recent meta-analysis including 18 pediatric studies found no consistent BMI change. However, increases in insulin requirements were reported in six out of eight included studies [35]. Several mechanisms may contribute to these findings. First, the intrinsic design of AID algorithms often results in more frequent corrective boluses and intensified insulin delivery, particularly to minimize postprandial hyperglycemia. This proactive insulinization strategy, although beneficial for achieving glycemic targets, may lead to higher overall insulin exposure. Second, insulin is an anabolic hormone, and sustained increases in insulin availability may promote adipose tissue deposition, especially if not balanced by adequate physical activity. Third, the greater dietary flexibility and reduced treatment burden associated with AID systems may inadvertently facilitate higher caloric intake, particularly in children and adolescents, who often report a sense of “freedom” from previous dietary constraints. The impact of advanced technologies on weight gain in people with diabetes raises concerns about potential long-term metabolic risks. In youth with T1D, obesity and overweight are increasingly recognized as emerging clinical issues, associated with higher cardiometabolic burden and greater insulin resistance [36]. However, BMI alone may not fully capture the nuances of these changes, as it does not distinguish between lean and fat mass. This limitation is particularly relevant in pediatric populations, where growth, maturation, and body composition shifts are dynamic and highly variable. Future studies should therefore integrate body composition assessments, longitudinal tracking of cardiovascular risk markers, and evaluation of lifestyle behaviors to better contextualize these findings. Such data will be critical to guide personalized recommendations, such as structured exercise programs and tailored nutritional counseling, aimed at balancing the metabolic benefits of AID systems with the prevention of undesirable weight gain.

This study has some limitations. The relatively small sample size may limit the generalizability of the findings, particularly when comparing outcomes between the two AID systems. Although we observed no significant differences, our sample may have been underpowered to detect subtle device-related variations, and this must be considered when interpreting the results. Furthermore, adherence and clinical outcomes with AID systems are known to be influenced by factors such as parental involvement, educational support, and socioeconomic status. As these variables were not systematically collected in our cohort, we could not account for their potential impact, and this represents an additional limitation. Moreover, the categorization of baseline HbA1c into subgroups, while offering clinically intuitive comparisons, may have led to a loss of information compared with treating HbA1c as a continuous variable. Finally, CGM metrics were derived from different cloud-based platforms, limiting access to raw data, although all metrics were calculated according to standardized consensus definitions. Nonetheless, the monocentric design represents a methodological strength, as it ensured all participants received uniform multidisciplinary education, minimizing educational bias.

## 5. Conclusions

Our study confirms that transitioning from MDI to AID therapy leads to early, significant, and sustained improvements in glycemic control in youth with T1D. Benefits are most pronounced in those with suboptimal baseline control. AID systems should be considered for all eligible pediatric patients, in accordance with prescription guidelines. However, to prevent undesirable increases in BMI, AID use needs to be complemented by lifestyle strategies, including regular physical activity and healthy dietary habits. Future studies with larger sample sizes and longer follow-up, focusing on body composition and cardiovascular risk factors in AID users, are warranted to provide a deeper understanding of the potential impact of these devices in youth with T1D.

## Figures and Tables

**Table 1:** Comparison of main glucose metrics at baseline, after 15 days of automated insulin delivery therapy and at 6 months. Data are expressed as mean values and standard deviation.

	Baseline	*p* ^a^	15 Days	*p* ^b^	6 Months	*p* ^c^
TIR (%)	62 ± 15.3	<0.001 *	76.7 ± 8.4	0.253	75.6 ± 9.1	<0.001 *
TITR 70–140 mg/dL (%)	39.8 ± 13	<0.001 *	53.9 ± 10.4	0.130	52 ± 10.6	<0.001 *
TAR > 180 mg/dL (%)	34.8 ± 15.8	<0.001 *	20.3 ± 8.5	0.162	21.7 ± 9.7	<0.001 *
TAR 180–250 mg/dL (%)	23.3 ± 8	<0.001 *	16.4 ± 6.7	0.183	17.4 ± 7.5	<0.001 *
TAR > 250 mg/dL (%)	11.7 ± 9.8	<0.001 *	3.9 ± 3.5	0.270	4.3 ± 3.9	<0.001 *
TBR < 70 mg/dL	3 ± 3.6	0.515	3 ± 2.6	0.190	2.6 ± 2.5	0.180
TBR 54–70 mg/dL (%)	2.6 ± 3	0.389	2.5 ± 1.9	0.167	2.2 ± 1.9	0.088
TBR < 54 mg/dL (%)	0.4 ± 0.8	0.215	0.6 ± 0.9	0.381	0.5 ± 0.8	0.516
GRI	43 ± 21.6	<0.001 *	24 ± 8.7	<0.001 *	27.5 ± 10.3	<0.001 *
GMI (%)	7.2 ± 0.8	<0.001 *	6.7 ± 0.3	0.067	6.8 ± 0.4	<0.001 *
Mean sensor glucose (mg/dL)	163.4 ± 26.2	<0.001 *	141.3 ± 11.9	0.035	144.9 ± 14.9	<0.001 *
CV (%)	36.9 ± 6.4	0.005	35.1 ± 6.1	0.344	34.2 ± 6.1	<0.001 *

^a^ Comparison between baseline and 15 days after AID; ^b^ comparison between 15 days and 6 months; ^c^ comparison between baseline and 6 months. CV: coefficient of variation; GMI: glucose management indicator; GRI: Glycemia Risk Index; SD: standard deviation; TAR: time above range; TBR: time below range; TIR: time in range; TITR: time in tight range. * significant *p*-value.

**Table 2:** CGM metrics and insulin data comparison between Medtronic MiniMed™ 780G (MM780G) and Tandem t:slim X2™ with Control-IQ (CIQ) users after 15 days and 6 months of use. Data are expressed as mean and standard deviation.

	15-Day Use			6-Month Use		
	MM780G	CIQ	*p*-Value	MM780G	CIQ	*p*-Value
TIR (%)	77.6 (9.6)	76.0 (7.4)	0.422	75.9 (9.5)	75.4 (8.7)	0.816
TITR (%)	54.6 (11.1)	53.4 (9.8)	0.645	52.6 (8.5)	51.5 (11.9)	0.640
TAR (%)	20.1 (9.9)	20.4 (7.3)	0.896	22.0 (9.9)	21.5 (9.5)	0.808
TAR1 (%)	17.1 (8.4)	15.8 (5.0)	0.426	18.9 (8.5)	16.3 (6.3)	0.127
TAR2 (%)	3.0 (3.2)	4.5 (3.5)	0.068	3.1 (2.9)	5.2 (4.3)	0.019 *
TBR (%)	2.2 (2.5)	3.7 (2.5)	0.015 *	2.0 (2.0)	3.1 (2.7)	0.057
TBR1 (%)	1.9 (2.0)	2.9 (1.7)	0.034 *	1.8 (1.6)	2.4 (2.0)	0.141
TBR2 (%)	0.3 (0.6)	0.8 (1.0)	0.031 *	0.3 (0.5)	0.7 (0.8)	0.021 *
GRI	21.8 (8.9)	25.5 (8.2)	0.129	25.2 (9.5)	29.2 (10.6)	0.475
GMI (%)	6.7 (0.3)	6.7 (0.3)	0.525	6.7 (0.3)	6.8 (0.4)	0.436
Mean SG (mg/dL)	140.1 (10.6)	142.2 (12.7)	0.468	143.4 (12.0)	145.9 (16.7)	0.467
CV (%)	33.3 (5.4)	36.3 (6.3)	0.035 *	32.2 (4.8)	35.6 (6.5)	0.016 *
Insulin TDD (IU/kg)	0.9 (0.3)	0.9 (0.3)	0.899	0.8 (0.2)	0.8 (0.2)	0.541
Basal delivery (%)	46.1 (8.9)	54.7 (8.6)	<0.001 *	40.7 (7.6)	55.1 (9.6)	<0.001 *
Autocorrection boluses (%)	33.1 (11.2)	17.2 (10.3)	<0.001 *	34.3 (10.7)	17.8 (12.1)	<0.001 *
AutoMode use (%)	98.4 (5.0)	92.6 (5.9)	<0.001 *	97.3 (4.7)	94.1 (5.5)	0.011 *

CIQ: Control-IQ; CV: coefficient of variation; GMI: glucose management indicator; GRI: Glycemia Risk Index; MM780G: MiniMed 780G; TAR: time above range; TBR: time below range; TIR: time in range; TITR: time in tight range; TTD: total daily dose. * significant *p*-value.

## Data Availability

The data presented in this study are available on request from the corresponding author.
